# Nurse educators’ experiences with student incivility: a meta-synthesis of qualitative studies

**DOI:** 10.3352/jeehp.2020.17.23

**Published:** 2020-08-11

**Authors:** Eun-Jun Park, Hyunwook Kang

**Affiliations:** 1 Department of Nursing, Konkuk University, Chungju, Korea; 2 College of Nursing, Kangwon National University, Chuncheon, Korea; Hallym University, Korea

**Keywords:** Education, Incivility, Qualitative research, Students, Systematic review

## Abstract

This study aimed to synthesize the best available qualitative research evidence on nurse educators’ experiences with student incivility in undergraduate nursing classrooms. A meta-synthesis of qualitative evidence using thematic synthesis was conducted. A systematic search was performed of 12 databases for relevant literature published by March 31, 2019. Two reviewers independently conducted critical quality appraisals using the checklist for qualitative research developed by the Joanna Briggs Institute. Eleven studies that met the inclusion criteria were selected for review. From the pooled study findings, 26 descriptive themes were generated and categorized into the following 5 analytical themes: (1) factors contributing to student incivility, (2) management of student incivility, (3) impact: professional and personal damage, (4) impact: professional growth, and (5) initiatives for the future. Many nurse educators became confident in their role of providing accountability as both educators and gatekeepers and experienced professional growth. However, others experienced damage to their personal and professional life and lost their motivation to teach. Nurse educators recommended the following strategies for preventing or better managing student incivility: institutional efforts by the university, unified approaches for student incivility within a nursing program, a faculty-to-faculty network for mentoring, and better teaching and learning strategies for individual educators. These strategies would help all nurse educators experience professional growth by successfully preventing and managing student incivility.

## Introduction

### Rationale

Respect and care are the essence of nursing [1]. Qualified nurses must demonstrate the value of human respect not only to patients, but also to their colleagues and other professionals. Nurse educators are responsible for ensuring that new nurses are equipped with these traits. The absence of respect in interpersonal interactions can lead to incivility [2]. Academic incivility is defined as speech or actions that violate the norms of mutual respect in educator–student interaction [3]. Unfortunately, the prevalence of uncivil behavior from students toward faculty (student incivility) seems to be growing in nursing education [4]. For example, a large number of Canadian nursing students experienced or witnessed uncivil behaviors such as arriving late for class (93.6%), holding conversations in class (86.2%), leaving class early (80.9%), general taunts or disrespect to faculty (69.2%), using a computer during class for purposes not related to the class (64.5%), or making disapproving groans (50.9%) [5]. Aul [6] concluded that nurse educators have concerns about this situation, since an uncivil student is more likely to become an uncivil nurse, who may threaten patient safety and jeopardize the workplace environment.

It is unclear whether student incivility occurs more often at nursing schools than in other disciplines. However, regardless of its relative severity, it is crucial to address student incivility since respect is a core value of the nursing profession. Therefore, nurse educators and researchers are paying increasing attention to student incivility [7], which has been studied in many countries, including China [8], Indonesia [9], Iran [10], South Korea [11], Oman [12], the United Kingdom [13], and Canada [14], and the United States [15,16]. Some types of uncivil student behaviors can be annoying or disturbing, such as being late for class, acting bored in class, or having side conversations. However, serious forms of incivility such as intimidating or threatening student behaviors toward nurse educators have also been reported [17].

The ability to prevent or minimize student incivility requires a complete understanding of the phenomenon. Recently, the number of nursing studies of academic incivility has increased, including qualitative, quantitative, and mixed-method studies [18]. Qualitative studies of academic incivility allow a deeper understanding of stakeholder experiences of incivility. However, the ability to generalize the experiences of the participants in an individual qualitative study is poor; therefore, a synthesis of qualitative studies is recommended when enough relevant evidence has been gathered. Of the several methods typically used for qualitative synthesis, this study selected thematic synthesis considering the different designs of primary qualitative studies, the largely descriptive evidence in the primary studies [19], and the methodological rigor of thematic synthesis [20].

While student incivility also occurs in clinical settings and online learning forums, academic institutions and classrooms are the contexts of interest in this study. A classroom is a place where nurse educators frequently interact with students face-to-face and serve as role models for professional behavior [21]. Hence, this study focused on student incivility occurring face-to-face in classrooms on campus.

### Objectives

The objective of this meta-synthesis was to expand our understanding of the experiences of nurse educators with student incivility in undergraduate nursing classrooms by synthesizing evidence from relevant qualitative studies.

## Methods

This review was prepared using the Preferred Reporting Items for Systematic Reviews and Meta-Analyses guideline [22]. The thematic synthesis method developed by Thomas and Harden [20] was used to synthesize the findings of qualitative studies.

### Eligibility criteria

We selected literature based on the types of participants (P), phenomena of interest (I), context (Co), and types of study, as presented in Table 1. A qualitative study was eligible for inclusion if it addressed student incivility (I) toward nurse educators (P) in undergraduate nursing classrooms (Co). This review included studies published up to March 31, 2019 with no limitation regarding language. Research participants were included if they were teaching or had taught undergraduate nursing students and had experienced student incivility, regardless of job title, tenure, or full-time employee status. A qualitative study was considered to deal with the phenomenon of student incivility if it presented a definition of student incivility consistent with that used in this review. Literature was excluded if it reported observations rather than self-reported experiences of student incivility, or reported a summary of qualitative data obtained from structured questionnaires. We also excluded studies that focused on only academic dishonesty, rather than student incivility in general. Some may argue that academic incivility includes academic dishonesty [23]. However, academic incivility mainly concerns interpersonal interactions, while academic dishonesty focuses on the ethical standards required in the process of pursuing knowledge and truth, such as cheating on examinations or plagiarism.

### Information sources

The literature search was conducted from April 1 to July 31, 2019. Twelve electronic databases were used for the literature search, including PubMed, CINAHL, Embase, Scopus, Web of Science, and ProQuest Databases (ABI/INFORM, Education, Education Source, ERIC, Psychology, Social Science, and Sociology). For searching the gray literature, we used ProQuest Dissertation and Thesis Global, Google Scholar, OpenGrey, and Deep Blue Library.

### Search

The 2 reviewers first independently searched PubMed and CINAHL and compared their search results in order to reach a consensus about the search strategy to apply to all of the databases. Through consultation with a professional librarian, the search strategy was confirmed. The keywords for the database search included “nursing faculty,” “nursing student,” “incivility,” and “qualitative study” in the combination of MeSH (Medical Subject Headings) terms, main subject terms, title words, and text words, which were combined using the “AND” and “OR” Boolean operators. The search strategy and keywords used for searching PubMed are presented in Table 2. We also conducted a manual search of the bibliographies of selected articles and content lists of the following relevant journals: *Nurse Education Today*, *Nurse Education in Practice*, *Journal of Nursing Education*, and *Qualitative Research*.

### Study selection

First, the 2 reviewers screened titles and abstracts according to the eligibility criteria after removing duplicate papers. The full texts of the included abstracts were then obtained and the 2 reviewers independently reviewed them to determine their eligibility for the synthesis of qualitative studies. Disagreements regarding paper inclusion were resolved by discussion.

### Data collection process and data items

The 2 reviewers individually read the primary studies and transcribed the study characteristics including study aims, study design, participants, data collection method, and findings. By cross-checking and discussing them, we reached a common understanding of the context of the studies. Before data extraction, the 2 reviewers independently read and repeatedly discussed the findings and discussion sections of the 11 studies that were finally included.

### Risk of bias

We used the checklist for qualitative research developed by the Joanna Briggs Institute (JBI) for quality appraisal [24]. We chose the JBI checklist because it focuses on congruity and comprehensively addresses the validity of qualitative studies [25]. The JBI checklist assesses the methodological quality of qualitative studies and the extent to which they address possible biases in design, conduct, and analysis. The JBI checklist consists of 10 items rated using 4 options (yes, no, unclear, or not applicable), and assesses whether a study was conducted congruently with appropriate methodology. We independently performed quality appraisal of the 11 finally selected studies and compared the results. When disagreements occurred, we reread the study report and discussed until reaching a consensus.

### Summary measures

Since this review is a synthesis of qualitative studies, a summary of measurements is not applicable.

### Synthesis of results

Before starting data synthesis, the 2 reviewers read and discussed the findings of the selected studies for data familiarization. When the reviewers reached a mutual understanding of the primary findings, the first step of thematic synthesis was undertaken by 1 reviewer (P.E.). Key concepts were extracted through line-by-line coding in the Results and Discussion sections of the selected studies using the MAXQDA software ver. 11 (VERBI GmbH, Berlin, Germany). The extracted codes were converted into a Microsoft Excel (Microsoft Corp., Redmond, WA, USA) file and reviewed by the other reviewer (K.H.).

In the second step, the reviewers independently grouped the free codes according to their similarities and differences. By agreement between the reviewers, descriptive themes were assigned to 26 groups of free codes. Lastly, the reviewers individually developed analytical themes by synthesizing descriptive themes, which were finalized through iterative discussions. Analytical themes consist of an interpretation beyond the meaning of the original studies [20]. The reviewers inferred nurse educators’ experiences through the descriptive themes in the context of nursing education. For example, 3 descriptive themes (accountability for the professional development of students, accountability as a gatekeeper, and moving forward to professionalism as an educator) related to the positive impacts of managing student incivility among nurse educators. Five analytical themes were generated regarding nurse educators’ experiences of student incivility. To validate that our synthesis was closely connected with the original data, we re-read the selected studies, applying the themes retrospectively to the primary codes and participants’ quotes.

### Additional analyses

No additional analyses were conducted for this review.

## Results

### Study selection

**Fig. 1.**
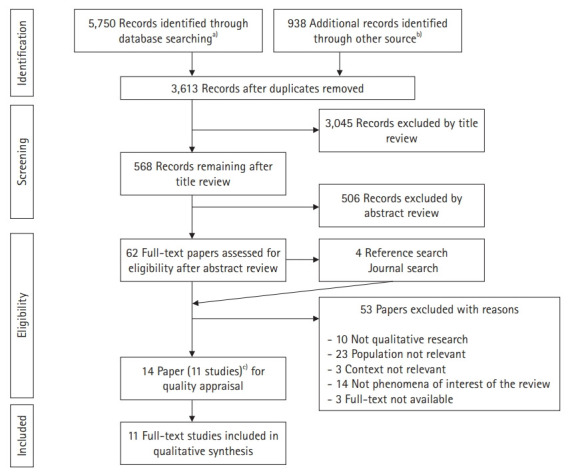
Flowchart of the study selection process. ^a)^PubMed 57+CINAHL 1,275+EMBASE 82+Web of Science 57+SCOPUS 38+ProQuest Education 1,353+ProQuest Psychology 1,044+ProQuest Education Source 513+ProQuest Social Science 462+ABI/INFORM 532+ProQuest Sociology 333+ERIC 4. ^b)^Google Scholar 3+ProQuest Dissertation and Thesis Global 934+OpenGrey 0+Deep Blue Library 0. ^c)^2 Included dissertation were published to 2 journal articles and 1 journal article.

Our literature search identified 6,688 papers that were potentially appropriate for inclusion in this review. The removal of duplicates across the databases left 3,613 papers, and a review of their titles and abstract yielded 62 articles for full-text reviews. An analysis of the reference lists of these 62 papers and a manual search added 5 more articles for full-text reviews. The full-text reviews of these 67 papers for eligibility resulted in 53 papers being excluded. The remaining 14 papers included 6 dissertations and 8 journal articles. Among them, 3 journal articles [17,26,27] were published from 2 doctoral dissertations [15,28], and thus 11 studies were finally included in the qualitative synthesis after quality appraisal. We reviewed both the dissertations and journal articles if applicable because the dissertations provided richer descriptions of the study findings than the corresponding journal articles. Fig. 1 shows the full literature search process.

### Study characteristics

The 11 studies included in the synthesis were conducted from 2003 to 2017, with 9 of the studies conducted during 2011–2017 (Table 3). Five of the 11 studies were conducted in the United States, 3 in Iran, 1 in the United Kingdom, 1 in Canada, and 1 in South Africa. The participants in all of the studies were either full-time or part-time nurse educators who experienced student incivility. The number of participating educators ranged from 9 to 21, and their genders were mixed in 5 studies, while 4 studies only included women, 1 study only included men, and gender information was not provided in the remaining study. The total number of participating nurse educators was 150. The most common research method was phenomenology (n=5), and data were collected by face-to-face individual interviews in all of the studies.

### Risk of bias

All 11 studies showed congruence between the research methodology and the research objectives (Q2 of the JBI checklist), data collection methods (Q3), the representation and analysis of data (Q4), and the interpretation of the results (Q5). Study participants and their voices were well represented (Q8) and closely related to the results and conclusions (Q10) in all the studies. For 6 studies, it was unclear whether congruity existed between the philosophical perspective and the research methodology (Q1). Eight studies clearly addressed the cultural and theoretical backgrounds of the authors (Q6) and described how potential influences of their values or backgrounds on the analysis student incivility were examined and handled (Q7). The results of the quality appraisal are presented in Table 4.

### Synthesis of results

Five analytical themes emerged from the 26 descriptive themes as presented in Table 5: (1) factors contributing to student incivility, (2) management of student incivility, (3) impact: professional and personal damage, (4) impact: professional growth, and (5) initiatives for the future.

#### Factors contributing to student incivility

Nurse educators identified the following common factors contributing to student incivility related to the students, the educators, the universities, or societies: stressors, intellectual and academic immaturity, entitlement and the consumerist mentality of the students themselves, poor teaching or classroom management of the lecturers, the university culture siding with students, and generational cultural differences including a lack of decorum. Nursing students were exposed to many stressors associated not only with academic performance, such as dissatisfaction with grades [14,15,29,30] and fear of failure in the academic program [14,31,32], but also with nonacademic activities such as work or family issues. These multifaceted stressors increased the possibility of uncivil behavior. Diverse forms of intellectual and academic immaturity [30] were also identified, such as academic unpreparedness [31], a lack of maturity [14], inappropriate understanding of faculty roles [29], and a lack of interest in learning [33]. Moreover, the educators perceived that students considered nursing education and educators to be a commodity and service providers, respectively, which resulted from the lens of consumerism [14,16]. The students felt that they had a business relationship with educators, since they paid tuition. The students thus believed they were entitled to ask for anything as paying consumers, and that educators should meet their needs [30]. Students with this mentality would not share the responsibility for learning with educators and tended to be dissatisfied with the demanding learning process.

Poor teaching practices of educators, such as disorganized course design or unexpected changes in the due dates for assignments, were believed to contribute to incivility [13,27,28,32]. The current university culture also negatively influenced incivility, because the university administration often sided with students rather than educators when disciplining uncivil students [14,28,32]. The cultural characteristics of the young generation increased student incivility. Nurse educators reported that the young generation has been raised in a society that lacks decorum or courtesy, making uncivil behaviors more acceptable than before. A high availability of communication technology facilitated the avoidance of personal contact, providing students with fewer opportunities to learn social skills than their educators had [14,28,30].

#### Management of student incivility

A wide range of uncivil student behaviors was exhibited [16,29]. Common behaviors included side conversations during classes [16,28], being late to class or leaving early [16,29], and arguing during test reviews [15,28,30]. More serious types of uncivil behavior like verbal or nonverbal threats and aggression [13,15,16,28,29] were also reported. The scope of student incivility was perceived differently by individual faculty members. For example, some faculty members considered that using smartphones during classes represented incivility [13,28], whereas other faculty members considered smartphone use to be an information-seeking behavior that could promote student learning [30]. Uncivil behaviors were considered distant from nursing values, and the educators were shocked by their students because they had never expected that students could show such uncivil attitudes or behavior toward their professors.

Educators managed instances of incivility with various approaches depending on their severity and circumstances, ranging from intentionally ignoring them or responding with a delay [32], making an indirect warning [10,32] and direct warning [10], to initiating formal disciplinary actions [10]. Important principles in addressing incivility were to maintain a courteous attitude toward uncivil students [15] and to maintain a professional boundary between the educator and the students [16]. The educators were not necessarily overly friendly or distant with students. Maintaining professional boundaries and demonstrating mutual respect is the base of a healthy relationship between both parties [14,16,29]. Uncivil behavior would be exacerbated if this boundary was breached or if an educator failed to show respect when interacting with a student.

While addressing incivility, educators were repeatedly ignored and experienced a lack of support from the university administration, which often created distrust of university administrators [14,15]. Many administrators were reluctant to support the educators and were likely to trivialize incidents of incivility. The educators were frustrated and felt alone when administrators refused to provide support and hindered disciplinary steps for uncivil students [16,28].

#### Impact on educators: professional and personal damage

The educators experienced a physical toll, emotional turmoil, and psychological distress during or after incidents of student incivility [15,28], with the severity and duration varying markedly from minor or temporary to traumatic or lasting for months or even years. Uncivil students often falsely accused educators publicly even in the absence of evidence, which consequently damaged the reputations of educators and increased their psychological distress [13,28,29]. Uncivil behaviors by students damaged educators’ self-esteem and confidence, often resulting in psychological trauma [15]. While the educators wondered why students behaved in that way in the self-reflection process, they often doubted that they had caused the incivility [15,16,28]. They questioned their aptitude or capability as educators, and were at risk of self-blaming [16]. They also had to spend long hours discussing and documenting the incidents, taking up time that they could otherwise have used for preparing lectures [15,28]. If they judged that their safety could be threatened by incivility, they often changed their teaching strategies to avoid confrontations with students [28]. When they were unable to manage a difficult incident, the educators became demotivated and their job satisfaction was negatively affected to varying degrees [29]. Even worse, a small number of educators left the education field due to experiences of incivility [15,16,28-30].

#### Impact on educators: professional growth

Through the experience of addressing incivility, the educators grew as educators and became more professional. As educators, they believed that they had to make the students know that uncivil behaviors were unacceptable and had to correct those behaviors for students’ professional development [14,30]. Nurse educators felt that they were also gatekeepers who facilitated competent students smoothly entering the nursing profession, while preventing those who were unprepared from doing so [30]. They were worried about the possible danger to patients or the entire nursing workplace when uncivil students become nurses without learning the value of civility [16,28,29]. Experiences of effectively managing uncivil students often helped educators to improve their teaching strategies and relationships with students [14,30].

While struggling with student incivility, a majority of educators were willing to stay in the education field due to numerous positive experiences with students. Educators came to have a better understanding of how to deal with students by listening to them while they managed incidents of uncivility [10,16]. They also learned the importance of respect in their relationship with students [30]. Experiences of the effectiveness of early interventions for incivility made them more proactively manage similar situations [16].

#### Initiatives for the future

Educators shared strategies that would be helpful to prevent or cope with student incivility, and these were grouped into 4 categories. They emphasized that universities needed to develop a code of conduct and to provide faculty members with training on student incivility [14,29]. Training programs may include providing knowledge about uncivil behaviors, conflict resolution, and strategies for managing classroom incivility. Providing counseling services for faculty members who encountered incivility was also beneficial [28]. Nurse educators suggested that the nursing programs should develop unified approaches for responding to uncivil student behavior [10,14], because inconsistent faculty responses may trigger further uncivil behavior. Uncivil behavior by students needs to be considered as problems of the program, rather than the involved faculty member’s personal issue [10].

Support from the program’s faculty-to-faculty network effectively alleviated educators’ emotional or professional distress while managing an incident. Experienced faculty provided effective strategies for new faculty in coping with different uncivil behaviors. Educators commonly described that they felt supported and validated when their colleagues shared their experiences and provided advice regarding how to manage student incivility [10,14,16]. Novice faculty members were particularly vulnerable to damage from uncivil student behaviors [16], and so they need to communicate with experienced faculty members on a regular basis in order to adequately manage student incivility [10]. In order to avoid conflict with students that may trigger incivility, educators emphasized the importance of effective teaching strategies. Often, they suggested that the syllabus needed to indicate limitations and allowances in the classroom as specifically as possible [28,30]. Promoting friendly relationships with students and engaging students in class activities were also effective for decreasing uncivil behaviors [10,30,32].

## Discussion

### Summary of evidence

Consistent factors contributing to student incivility were reported across the selected studies. University students perceived themselves as consumers of education services and felt entitled to obtain high grades and a degree in return for paying tuition. At the same time, universities recognized students as their primary consumers, which resulted in students having power and exhibiting entitlement and a passive attitude to learning [34,35]. This is very worrisome because students with a stronger consumer orientation toward education tended to behave more uncivilly and to perform more poorly academically, as shown by low grades [35]. Students should be considered as partners in higher education, rather than as consumers; as such, they should share power in the classroom and receive corrective feedback from educators [36]. Their voices are valuable to enhancing the quality of education, but their responsibility for learning should not be lessened.

In the processes through which nurse educators managed student incivility, a lack of administrative support was a major obstacle. The voices of educators were not properly considered when making decisions about incivility incidents. One of the possible reasons for reluctance among administrators is their conflict of interest with faculty. Administrators are likely to be concerned about the possible damage to the public image of a school and any loss of students and tuition income resulting from disciplining an uncivil student [14]. However, the reputation of a nursing school will also suffer if the irresponsible behaviors of graduates threaten patient safety. Administrators need to be more proactive in handling student incivility by listening to and providing advice to faculty in a supportive way. Support from administrators is a critical predictor of the intention of faculty to remain in academia [37], and leadership from administrators is critical in creating and maintaining a civil culture [38].

Experiences of managing student incivility resulted in professional growth for some educators. Nurse educators came to be confident in their roles as educators and gatekeepers. Unlike educators in general, nurse educators play gatekeeper roles in protecting both individual patients and the nursing profession as a whole. They felt empowered by knowing the best approach to prevent and manage student incivility, such as being proactive in managing student behavior, holding students accountable for their behaviors, and keeping professional boundaries in their relationships with students. By being proactive, educators can notice and intervene in possible incivility early enough to avoid serious problems. Furthermore, nurse educators realized the importance of teaching professional behaviors and civility. Students need to learn how their behaviors can be perceived as being uncivil depending on the context, and how they can behave in a respectful way even in a conflict situation using assertive communication skills. Civility education for students needs to be better integrated in nursing curricula [38], and should be evaluated in nursing school accreditation measures.

This review found various strategies for the prevention and management of student incivility. Institutional initiatives have been consistently suggested in the literature, including developing an incivility policy and a code of conduct, raising awareness of incivility, and training faculty in classroom management [6,39]. The severity of incivility was subjectively assessed according to individuals’ beliefs or previous experiences [14]. Furthermore, there were also cultural differences regarding incivility; in Iran for instance, a student walking ahead of a professor or being self-assertive was considered uncivil [33,40]. These findings warrant open communication and a consensus regarding professional nursing behaviors in each culture. Facilitating clear communication about both undesirable and desirable behaviors among the various stakeholders should be prioritized. This can be initiated by announcing a policy and code of conduct at the university level. At a classroom level, a code of conduct needs to be developed with student input, and then students should sign the code of conduct as a contract. Moreover, behavior-related policies and a zero-tolerance policy should be stated in the syllabus and communicated in the first class [2,39].

A unified approach to incivility and a faculty-to-faculty support network were unique strategies identified in this study. It is important to send a consistent message to students about both undesirable and desirable behaviors at the level of nursing programs. If both nursing faculty and students participate in developing a code of conduct, a common understanding about expected civil behaviors can be established, and different faculty members can provide consistent approaches to student incivility. To ensure a consistent response, it is preferable for a dean or a chair of a nursing program to announce the code of conduct of the school before individual faculty members reinforce it in their classes. In addition, a faculty-to-faculty network was emphasized for providing emotional support and seeking advice from a mentor. Nurse educators need to share successful experiences of incivility prevention or management [41].

The reviewers identified research gaps in the current literature. Research on faculty–student boundaries is lacking in nursing. Nurse educators readily identified a boundary violation at the moment that an incivility event occurred. Maintaining professional faculty–student boundaries seems to be a prerequisite for civil interactions and a pedagogical relationship [16]. When a boundary is breached, the power of the faculty member as an expert is lessened, and accordingly the probability of student incivility is likely to increase [42]. Further studies are therefore needed to identify the appropriate distance that should be maintained between faculty and students, and how different boundaries influence faculty–student interactions and learning outcomes. Synthesizing qualitative research on faculty-to-student incivility is also recommended to provide a comparable counterpoint to the present findings on student-to-faculty incivility. Both faculty and students are responsible for cultivating a civil culture in academia, and therefore experiences of incivility need to be heard from both sides.

### Limitations

The trustworthiness or credibility in a meta-synthesis of qualitative research could be at risk for various reasons. Regarding the trustworthiness of the study, 2 reviewers independently performed all of the study processes and any discrepancy between the reviewers was rigorously discussed until agreement was reached. To ensure that the synthesis faithfully reflected the interpretations of the primary authors, all of the thematic codes were retraced after a synthesis was performed. We also performed a systematic and thorough sampling with clearly defined inclusion criteria and maintained an audit trail throughout the study process. Not all of the retrieved studies were of the highest quality. However, it is an inherent limitation of meta-syntheses that their findings are limited by the quality and interpretations of the original studies [43].

### Conclusions

Civility is a critical virtue in health professionals and nurses, and therefore, student incivility issues should be discussed among educators without delay. This review found that the management of student incivility was challenging for nurse educators, bringing about distress in their personal life and threatening their professional status. Nevertheless, while experiencing student incivility, some nurse educators moved forward as educational professionals by focusing on accountability, while others compromised their teaching strategies and lost their motivation to teach. Nurse educators recommended the following strategies for preventing or better managing student incivility: institutional efforts by the university, unified approaches for student incivility within a nursing program, a faculty-to-faculty network for mentoring, and better teaching and learning strategies for individual educators. These strategies are practical and would help all nurse educators experience professional growth by successfully preventing and managing student incivility.

## Figures and Tables

**Table 1.** Inclusion criteria and search terms used for the review

Variable	Description	Search terms
Participants	Nurse educators or faculty members	Nursing faculty, nurse faculty, nursing educator, nurse educator
Phenomenon of interest	Incivility of nursing students	Incivility, aggression, aggressive behavior, bullying, dishonesty, disruptive behavior, rudeness, mobbing, non-sexual harassment, uncivil behavior, violence
		Nursing student, nurse student, student nurse, pupil nurse
Context	Undergraduate nursing classrooms	No limit
Type of study	Qualitative studies	Qualitative study, qualitative data, phenomenology, ethnography, grounded theory, hermeneutics

**Table 2.** PubMed search strategy and results (as of May 21, 2019)

Key concepts	Search terms with a publication date limit “March 31, 2019”	Results
1. Nurse educator	Faculty, nursing [MeSH]	36,909
	Faculty, nursing [TW] OR faculties, nursing [TW] OR nursing faculties [TW] OR nursing faculty [TW]) OR nurse faculty [TW] OR nurse faculties [TW] OR nurse-faculty [TW] OR nurse educator [TW] OR nurse educators [TW] OR nursing educator [TW] OR nursing educators [TW]) OR nursing professor [TW] OR nursing professors [TW] OR nurse professor [TW] OR nurse professors [TW]	
2. Nursing student	Students, nursing [MeSH]	27,915
	Students, nursing [TW] OR pupil nurses [TW] OR student, nursing [TW] OR nurses, pupil [TW] OR nurse, pupil [TW] OR pupil nurse [TW] OR nursing student [TW] OR nursing students [TW]	
3. Incivility	Incivility [MeSH], aggression [MeSH], harassment, non-sexual [MeSH]	113,524
	Incivility [TW] OR rudeness [TW] OR uncivil behavior [TW] OR uncivil behaviors [TW] OR behavior, uncivil [TW] OR behaviors, uncivil [TW] OR workplace incivility [TW] OR incivility, workplace [TW] OR uncivil behavior [TW] OR uncivil behaviors [TW] OR disruptive behavior [TW] OR disruptive behaviors [TW] OR bullying [TW] OR violence [TW] OR mobbing [TW] OR dishonesty [TW] OR dishonest behavior [TW] OR dishonest behaviors [TW]	
4. Qualitative study	Qualitative research [MeSH], grounded theory [MeSH]	98,683
	Qualitative research [TW] OR research, qualitative [TW] OR qualitative study [TW] OR qualitative data [TW] OR grounded theory [TW] OR phenomenology [TW] OR hermeneutic [TW] OR ethnography [TW]	
5. Full search	1 AND 2 AND 3 AND 4	57

**Table 3.** Table caption

Serial no.	Author (year)	Country	Aims	Study methodology	Participants (gender)	Data collection		Findings
1	Luparell [15] (2003)^a)^, Luparell [17] (2004), Luparell [26] (2007)	USA	To explore how nursing faculty describe uncivil encounters with students in terms of the nature of the incidents and their effects on faculty	Critical incident technique; Flanagan’s (1954) method	21 Educators; 20 women and 1 man	Semi-structured interviews: face-to-face and telephone		Framework: incivility as a battle
			To present a framework for describing faculty experiences with incivility					Before the confrontation: escalating tensions and triggering events, diplomatic efforts
								On the battlefield: ambush, attacks, battlefield emotions, calling in reinforcements-and MEDIC!
								The aftermath: missing in action and costs of war, physical toll, injury to self-esteem and confidence, emotional toll and posttraumatic stress, time expenditure, financial costs, retreat and withdrawal
2	Olive [16] (2006)^a)^	USA	To explore the lived experience of nursing faculty members who have experienced extreme forms of nursing student incivility	Heidegger’s hermeneutic phenomenology	16 Educators; all women	Face-to-face interviews		Pattern: dwelling with a sense of being alone while moving towards understanding
								6 Themes: discovering student issues, perceiving potential for violence, engaging in a self-interpretive process, growing as a nurse educator, feeling alone as a gatekeeper, recognizing there’s something to be learned
3	Williamson [29] (2011)^a)^	USA	To explore the experiences and impact of incivility on nurse educators	Interpretive phenomenology, inductive thematic analysis	10 Educators; all women	Face-to-face interviews		7 Themes: uncivil experiences, nurse educators’ emotions, impact of incivility, addressing incivility, warning signs or contributing factors, prevention of incivility, incivility a growing problem
4	White [13] (2013)	England	To identify the means by which faculty working within post-1992 universities in England are being subjected to harassment by undergraduate students and to establish their explanation regarding the context of the harassment	Not reported	12 Educators; 6 women and 6 men	Interviews		3 Themes: verbal and task attack, personal attack, communication devices used to harass
5	Sprunk [28] (2013)^a)^, Sprunk et al. [27] (2014)	USA	To describe and understand the experiences and impacts among nursing faculty who encountered nursing student incivility	Husserl’s transcendental phenomenology, Colaizzi’s analysis	12 Educators; all women	Face-to-face or telephone interviews	6 Themes: variety of unacceptable student behaviors, time consuming, tarnished reputation, support is beneficial, harmful to health and well-being, questioning the future	
6	Sweetnam [14] (2014)^a)^	Canada	To explore the perceptions and lived experiences of full-time and part-time nursing faculty members teaching in university undergraduate nursing programs	Descriptive phenomenology, Colaizzi’s method	14 Educators; all women	Face-to-face interviews	8 Themes: the uncivil environment, behavior triggers, circumventing accountability, faculty stand alone, toll, concerns about the future of nursing, responsibility: a faculty imperative, getting by with a little help	
7	Vink et al. [31] (2015)	South Africa	To describe what nurse educators consider to be factors contributing to incivility among nursing students in South African nursing school	Qualitative descriptive design	11 Educators	Face-to-face interviews	3 Themes: academic factors, Psycho-pathological factors, Social factors	
8	Rad et al. [10] (2016)	Iran	To reflect strategies of Iranian educators in dealing with nursing students’ incivility	Qualitative content analysis	14 Educators; 6 women and 8 men	Face-to-face interviews	9 Themes: freedom in classroom, appropriate decision-making, authority, training through role-playing, friendship strategy, teaching-learning strategy, unification of educators regarding behavior management, interactive educational environment, self-reflection	
					8 Students; 4 women and 4 men			
9	Leech [30] (2017)^a)^	USA	To explore the lived experiences of male nurse educators and effect nursing student incivility has on pedagogy and job satisfaction	Heidegger’s hermeneutic phenomenology	9 Educators; all men	Face-to-face and Skype interviews	6 Themes: uncivil events, physical and emotional responses to student incivility, reasons for student incivility, actions to address incivility, the gatekeeper role, men mentoring men in nursing	
10	Masoumpoor et al. [33] (2017)	Iran	To determine the perceptions of nursing educators about students’ uncivil behavior	Conventional content analysis	11 Educators; 8 women and 3 men	Semi-structured interviews	3 Themes: disruptive behaviors affecting communication climate, disruptive behaviors affecting ethical climate, disruptive behaviors affecting learning climate	
11	Rad et al. [32] (2017)	Iran	To discover teachers’ and students’ experiences regarding incivility among students and to develop an approach to managing students’ incivility	Grounded theory methodology	20 Educators; 5 women and 15 men	Face-to-face interviews, post-interview comment sheets	4 Categories: deterioration of learning, dominant individual and organizational culture, guided democracy, movement, movement to professionalism	
					9 Students; 5 women and 4 men			

a) Doctoral dissertation.

**Table 4.** Results of critical quality appraisal using the Joanna Briggs Institute checklist

Author (year)	Q1	Q2	Q3	Q4	Q5	Q6	Q7	Q8	Q9	Q10	No. of ‘Y’
Luparell [15,17,26] (2003, 2004, 2007)	U	Y	Y	Y	Y	Y	Y	Y	Y	Y	9
Olive [16] (2006)	Y	Y	Y	Y	Y	Y	Y	Y	Y	Y	10
Williamson [29] (2011)	Y	Y	Y	Y	Y	Y	Y	Y	Y	Y	10
White [13] (2013)	U	Y	Y	Y	Y	U	U	Y	U	Y	6
Sprunk et al. [27,28] (2013, 2014)	Y	Y	Y	Y	Y	Y	Y	Y	Y	Y	10
Sweetnam [14] (2014)	Y	Y	Y	Y	Y	Y	Y	Y	Y	Y	10
Vink et al. [31] (2015)	U	Y	Y	Y	Y	U	Y	Y	Y	Y	8
Rad et al. [10] (2016)	U	Y	Y	Y	Y	Y	Y	Y	Y	Y	9
Leech [30] (2017)	Y	Y	Y	Y	Y	Y	Y	Y	Y	Y	10
Masoumpoor et al. [33] (2017)	U	Y	Y	Y	Y	Y	U	Y	Y	Y	8
Rad [32] (2017)	U	Y	Y	Y	Y	U	U	Y	Y	Y	7

Q1, congruity between philosophical perspective and methodology; Q2, congruity between methodology and research questions; Q3, congruity between methodology and data collection methods; Q4, congruity between methodology and representation and analysis of data; Q5, congruity between methodology and interpretation of results; Q6, statement locating the researcher culturally or theoretically; Q7, influence of the researcher on the research; Q8, adequate representation of participants’ voice; Q9, evidence of ethical approval; Q10, conclusions drawn from data analysis or interpretation; Y, yes, U, unclear.

**Table 5.** Findings of the synthesized translations

Analytical themes	Descriptive themes	Reference no. of source study	Supporting quotes
1. Factors contributing to student incivility	Stressors	[13,14,16,29-32]	“I feel in nursing, they have so much to do, so many assignments and activities, the required number of clinical and lab hours, their theory hours and their written exams… They have to work for gas money to even get to school…” [29]
	Intellectual and academic immaturity	[13,14,16,29,31-33]	“… when students enter the university as freshmen, they are still acting as high schoolers thinking that they can be free and relaxed here too; they treat people around them, their friends and professors the way they wish…” [32]
	Entitlement and consumerism mentality	[13,14,16,30]	“I have had many students that I feel like they just act as if we owe them everything in the world just because they’re a student and they pay tuition. They are just allowed to say and do whatever they want because they think it is a business relationship and they are the customer.” [24]
	Poor teaching or classroom management	[13,29,31,32]	‘Then also it can also be the attitude of the lecturers… because I think sometimes lecturers can also be very abrupt and you know behavior breeds behavior.” [31]
	University culture	[14,16,31,32]	“You feel as if there is this personal […] defaming that happens because you’ve been called in to the director’s office now twice for the same sort of nonsense. I do call it nonsense. I do think the culture at [the university] is one of pleasing students. It’s not about protecting faculty.” [14]
	Generational culture	[13,14,30,31]	“Yes, it is a big growing problem. I believe it has to do with our students… many of them are the generations of computers. They don’t have the social skills or the social manners that we were raised with.” [29]
2. Management of student incivility	A range of uncivil behaviors	[13-16,28-30,33]	“Before, I thought of a more disrespectful attitude… I now know that incivility can be more than just a bad attitude and smart remarks… it can make you feel unsafe.” [29]
	A sense of shock	[14-16,29]	“I was just flabbergasted… My first thought was I cannot believe they’re behaving this way… I was amazed…” [15]
	Applying various strategies to an incivility incident	[10-29,30,32]	“In other words, we use various methods. A single one is not working on everyone. You should know that in each term, you have a new version of students.” [10]
	Approaching uncivil students in a courteous manner as a role model	[10,14,15,32]	“… but here, I directly reminded the student of her inappropriate behavior on the basis of the university rules and regulations.” [32]
	Establishing professional boundaries	[10,14,16,29,33]	“We are still their instructors and we need to keep our relationship on a level where we can maintain that faculty/student relationship.” [29]
	Feeling alone without support	[14-16,28,31]	“I kind of feel like almost defeated. I felt almost victimized that it was allowed to go on without repercussion, and I felt faculty are almost a sitting target.” [28]
	Distrust of administration	[14-16]	“But it was—the most unsettling thing was that it was a group of my peers that allowed her to stay, …That other people weren’t looking at the profession like I was, they weren’t looking at the safety of others. They were more worried about law suits and money and their clients.” [16]
3. Impact: professional and personal damage	Threats to physical well-being	[15,28,30]	“I experienced all of that [troubling sleeping, headaches, decreased concentration], some short-term memory where I had things to pick up, something at the store, go meet someone, missing a whole lot of appointments.” [30]
	Emotional turmoil and psychological trauma	[13-16,28-30]	“There were rumors going around the university about me, like my partner was a prostitute and a drug dealer and that I condoned her lifestyle… lived off her…, that I didn’t have a PhD… Of course, it was a complete pack of lies.” [13]
	Damage to self-esteem and confidence	[10,13-16,28-31]	“I almost felt sick, I was rattled. I was rattled to the core. I took blame. I felt blame… I felt like it was my fault, because I allowed it to happen. How did I allow this to happen? How did I allow myself to be bullied? Like, what the heck’s wrong with you?” [14]
	Expenditure of time and money	[14,15,28]	“And we addressed it several times during the fol­lowing week, as we made arrangements to meet with her [the student] together… You know, maybe 16 hours of actual work, docu­menting, rehashing it, talking it over with supervisors, with the assistant dean, going over it… writing it up. At least 16 hours.” [15]
	Compromising teaching strategies for self-protection	[14,15,29]	“I will never give a student less than a B anymore, because it’s not worth it to me—my physical and mental, emotional health—to go through that in this system. I will never do it again.” [15]
	Demotivation and job dissatisfaction	[13-15,27,28,33]	“When things like that occur, it makes me unhappy in my job, I wonder why I am doing
			this, I could be doing lots of other things, I don’t have to put up with this…” [29]
4. Impact: professional growth	Accountability for the professional development of students	[10,14,16,32]	“I will continue, and I need to hold them[students] to accountability. And, — because I feel that I have a moral and ethical responsibility to society to provide and educate competent practitioners, and I will not compromise that.” [16]
	Accountability as a gatekeeper	[14,16,30]	“Now I'm thinking of this person wandering—going through her nursing education and now becoming a staff nurse. And, now I’m fearful of that person out taking care of patients—my parents, my family…” [16]
	Moving forward to professionalism as an educator	[15,16,29,32]	“… as our experiences increased, we were skillful in teaching the content; we started getting more familiar with how to treat students; we came to an understanding that we needed to value students, to keep good relations with them, to listen to their stories, and to value them…” [32]
5. Initiatives for the future	Institutional initiative	[14,29,30,32]	“We need more classes available to teach us how to deal with this type situation, not just at the moment, but how you react to the student after that point. What barriers do you need in the relationship to prevent incidences from happening again?” [29]
	Unified approach	[10,14,16]	“The class has been overall much better. But I think some of it is because it wasn’t just me. The entire level got behind me to say this isn't appropriate behavior and it needs to stop now.” [16]
	Faculty-to-faculty support network	[14,16,28,32]	“I think faculty need to know that they need to talk to other faculty when they’re dealing with these challenging students. One of the blessings that I had at both organizations is that I had faculty colleagues who were very experienced clinicians and offered me some wonderful sage advice. And, not all of them were nursing, by the way. Some of them were other disciplines.” [16]
	Improving teaching and learning strategies	[10,30,33]	“…We always go over the syllabus the very first day… I set the ground rules at the beginning of class… It is important they know where you stand and they need to follow the rules. When you have policies, enforce them.” [30]

## Supplementary materials

Supplement 1. The audio recording of the abstract.
